# Meroterpenoids from *Ganoderma* Species: A Review of Last Five Years

**DOI:** 10.1007/s13659-018-0164-z

**Published:** 2018-05-02

**Authors:** Xingrong Peng, Minghua Qiu

**Affiliations:** 1 State Key Laboratory of Phytochemistry and Plant Sources in West China, Kunming Institute of Botany, Chinese Academy of Sciences, Kunming, 650201 People’s Republic of China; 2 University of the Chinese Academy of Sciences, Beijing, 100049 People’s Republic of China

**Keywords:** *Ganoderma*, *Ganoderma* meroterpenoids, New structures, Biological activities

## Abstract

**Abstract:**

Meroterpenoids are hybrid natural products that partially originate from the terpenoid pathway. *Ganoderma* meroterpenoids (GMs) are a type of meroterpenoids containing a 1,2,4-trisubstituted phenyl and a polyunsaturated terpenoid part. Over last 5 years, great efforts have been made to conduct phytochemistry research on the genus *Ganoderma*, which have led to the isolation and identification of a number of GMs. These newly reported GMs showed diverse structures and a wide range of biological activities. This review gives an overview of new GMs from genus *Ganoderma* and their biological activities and biosynthetic pathway, focusing on the period from 2013 until 2018.

**Graphical Abstract:**

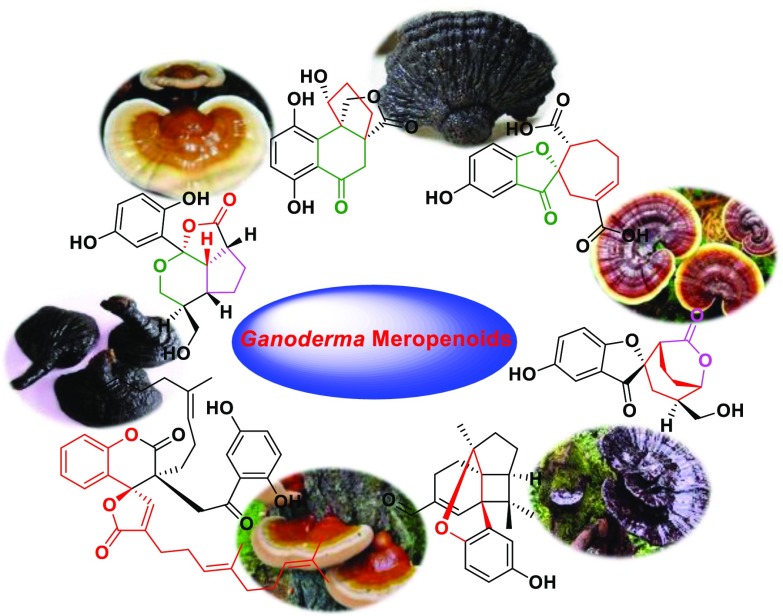

## Introduction

*Ganoderma* is a ganodermataceae (basidiomycete) white rot fungus, normally growing on woody plants and wood logs [1], and is used for medicinal purposes in China, Japan, and South Korea (Chinese Higher Fungi: 18 volumes). It was first recorded in the *Shennong’s Classic of Meteria Medica*, and classified as an upper-grade medicine in medical books [2]. About 78 species of *Ganoderma* are recorded in Chinese Higher Fungi, of which, *G. lucidum* and *G. sinense*, were found to be edible and medicinally-beneficial fungi, and were registered in Chinese Pharmacopoeia (2010 and 2015 edition). However, other species, such as *G. capense*, *G. cochlear,* and *G. tsuage*, also play an important part in traditional folk medicines. In addition, pharmacological studies have also involved the extract and chemical constituents of other species [3–5]. Until now, the chemical constituents and biological activities of 22 species of *Ganoderma* have been studied.

*Ganoderma* is rich in novel “mycochemicals”, including polysaccharide, triterpenoids, steroids, fatty acids, etc. Although polysaccharide is found to be one of the main bioactive constituents, its high molecular weight and complex structure limits its use in the drug market. Meanwhile, the small molecular constituents have played a significant role over the last 200 years in treating and preventing diseases, and are continuing to serve as important leads in modern drug discovery [6–11].

Since the discovery of ganomycins A and B [12], more than 100 aromatic meroterpenoids, derived by a hybrid of shikimic acid and mevalonic acid biogenetical pathway, were isolated from the genus *Ganoderma* (Ganodermataceae) [13]. *Ganoderma* meroterpenoids (GMs) have attracted increasing attention because they showed diverse structural skeletons and series of bioactivities, such as NO production inhibitory [14], anti-oxidant [15, 16], anti-allergic [17, 18], anti-fibrotic [19], anti-Acetyl cholinesterase (AChE) [20], cytotoxic [21], antimicrobial [12], and aldose reductase inhibitory activities [22]. As a result, chemists have synthesized polycyclic meroterpenoids by employing many steps [23–26].

Herein, we review the structure, bioactivities, and biosynthesis pathways of GMs from *Ganoderma* species to lay the foundation for the further research and provide the important sources for the development of lead compounds.

## Biosynthetic Pathway of GMs

The prenylation of aromatic compounds plays an important role in the natural product research because it not only gives rise to an astounding diversity of small molecular constituents in plants, fungi and bacteria, but also enhances the bioactivities and bioavailabilities of these compounds [27]. Aromatic prenyltransferase is the key enzyme for the prenylation of aromatic compounds. Meroterpenoids including ubiquinone, plastoquinone, menadione, vitamin E, prenylflavonoids, shikonin and prenylated alkaloids, are formed under prenyltransferase [28]. The analysis of the genome showed that abundant carbohydrate-active enzymes and ligninolytic enzymes were present in the *G. lucidum* genome [29]. All the meroterpenoids from *Ganoderma* consist of a 1,2,4-trisubstituted phenyl group and a polyunstaturated terpenoid parts, suggesting that lignin was degraded to phenyl group by the liginolytic enzymes of *Ganoderma*, and the terpenoid parts were further assembled under prenyltransferase.

## Chemical Structures and Bioactivities of GMs

A class of GMs, which had a 1,2,4-trisubtituted phenyl group connecting with C10 or C15 polyunsaturated side chain or polycyclic substructure, widely distributed in genus *Ganoderma*. According to the difference in their terpenoid parts, these GMs can be divided into three types.

### Chain-Contained GMs

**Fig. 1:**
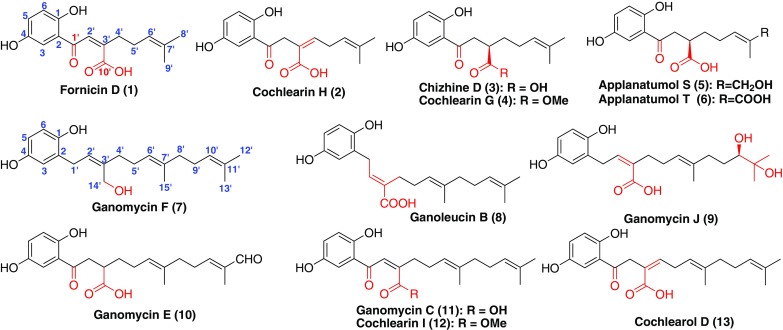
Structures of GMs with a 10-carbon or 15-carbon chain

Due to the presence of double bonds in terpenoid part, the redox reaction can take place in allylic position (Fig. 1, Table 1). Thus, compounds **1**–**6**, and **9–13** had a ketone carbonyl at C-1′ and a carboxyl or methyl ester at C-10′ or C-14′ [15, 16, 30–35]. Among them, compounds **2** and **13** existed positional isomerization of olefinic bond because of the shift of the double bond at C-2′ and C-3′ [30, 35], whereas, the reduction of the ∆^2^′^,3^′ in chizhine D (**3**), cochlearin G (**4**), applanatumols S, T (**5**, **6**) and ganomycin E (**9**) was occurred [30, 31, 34]. The C-14′ of ganomycin F (**7**) was connected to a hydroxyl group [16]. The $trans{ - }\Delta^{{ 2^{\prime } , 3^{\prime } }}$ of ganoleucin B (**8**) was isomerized to *cis* under conditions of enzyme or light [33]. The $\Delta^{{ 1 0^{\prime } ,11^{\prime } }}$ of ganomycin J (**9**) was oxidized to two hydroxyls. Fornicin D (**1**), cochlearins H, G, I (**2**, **4**, **12**) and ganomycin C (**11**) isolated from *Ganoderma cochlear*, as well as ganomycins F and E (**7** and **10**) gained from *G. capense*, showed significant anti-oxidant activities [15, 16, 30]. Compound **3** was isolated from *G. lucidum* and displayed weak renoprotective effect [31]. The biological assay of applanatumols S and T (**5**, **6**) from *G. applanatum* [32], and ganoleucin B (**8**) from *G. leucocontextum* didn’t show inhibitory activities against COX-1, COX-2, HMG-CoA reductase and *α*-glucosidase, respectively [33]. However, ganomycin J (**9**) from *G. lucidum* showed strong inhibitory activity against HMG-CoA reductase with an IC_50_ value of 30.3 μM [34].

**Table 1:** Name, source and their bioactivities of chain-containing GMs

Number	Name	Bioactivity	Source	Reference
**1**	Fornicin D	Antioxidant activity	*G. cochlear*	[15]
**2**	Cochlearin H	Antioxidant activity	*G. cochlear*	[30]
**3**	Chizhine D	Renoprotective effect	*G. lucidum*	[31]
**4**	Cochlearin G	Antioxidant activity	*G. cochlear*	[30]
**5**	Applanatumol S	Inhibitory activities against COX-1, COX-2	*G. applanatum*	[32]
**6**	Applanatumol T	Inhibitory activities against COX-1, COX-2	*G. applanatum*	[32]
**7**	Ganomycin F	Antioxidant activity	*G. capense*	[15]
**8**	Ganoleucin B	Inhibitory activities against HMG-CoA reductase and *α*-glucosidase	*G. leucocontextum*	[33]
**9**	Ganomycin J	Inhibitory activity against HMGs reductase (IC_50_: 30 μM), aldose reductase and α-glucosidase	*G. lucidum*	[34]
**10**	Ganomycin E	DPPH radical scavenging activity	*G. capense*	[16]
**11**	Ganomycin C	Antioxidant activity	*G. cochlear*	[15]
**12**	Cochlearin I	DPPH radical scavenging	*G. cochlear*	[30]
**13**	Cochlearol D		*G. cochlear*	[35]
**14**	(+)-Applanatumol U	Inhibitory activity against COX-1 and COX-2	*G. applanatum*	[32]
**15**	(+)-Chizhine E	Renoprotective effects	*G. lucidum*	[31]
**16**	(+)-Lucidulactone B		*G. lucidum*	[36]
**17**	(+)-Zizhine A	Renoprotective effects	*G. sinense*	[37]
**18**	(+)-Ganoleucin C	Inhibition against HMG-CoA reductase and a-glucosidase	*G. leucocontextum*	[33]
**19**	(+)-Chizhine F	Renoprotective effects	*G. lucidum*	[31]
**20**	(+)-Zizhine B	Renoprotective effects	*G. sinense*	[37]
**21**	(+)-Zizhine C	Renoprotective effects	*G. sinense*	[37]
**22**	(+)-Zizhine D	Renoprotective effects	*G. sinense*	[37]
**23**	(+)-Zizhine E	Renoprotective effects	*G. sinense*	[37]
**24**	(+)-Zizhine F	Renoprotective effects	*G. sinense*	[37]
**25**	(+)-Fornicin E	Renoprotective effects	*G. cochlear*	[16]
**26**	Chizhine A	Renoprotective effects	*G. lucidum*	[31]
**27**	Chizhine B	Renoprotective effects	*G. lucidum*	[31]
**28**	Chizhine C	Renoprotective effects	*G. lucidum*	[31]
**29**	(+)-Cochlearin B	Antioxidant activity	*G. cochlear*	[30]
**30**	(±)-Cochlearin D	Antioxidant activity	*G. cochlear*	[30]
**31**	(+)-Lingzhine E	Neural stem cell proliferation	*G. lucidum*	[38]
**32**	(+)-Applanatumol P	Inhibitory activity against COX-1 and COX-2	*G. applanatum*	[32]
**33**	(+)-Applanatumol Q	Inhibitory activity against COX-1 and COX-2	*G. applanatum*	[32]
**34**	(+)-Applanatumol R	Inhibitory activity against COX-1 and COX-2	*G. applanatum*	[32]
**35**	(±)-Ganocapensin A	Inhibitory activity against COX-1 and COX-2	*G. capense*	[16]
**36**	Ganocapensin B	Antioxidant activity	*G. capense*	[16]
**37**	(±)-Cochlearin E	Antioxidant activity	*G. cochlear*	[30]
**38**	Cochelarin F	Antioxidant activity	*G. cochlear*	[30]
**39**	Applanatumol Z1	Inhibitory activity against COX-1 and COX-2	*G. applanatum*	[32]
**40**	Cochlearol C		*G. cochlear*	[33]

**Fig. 2:**
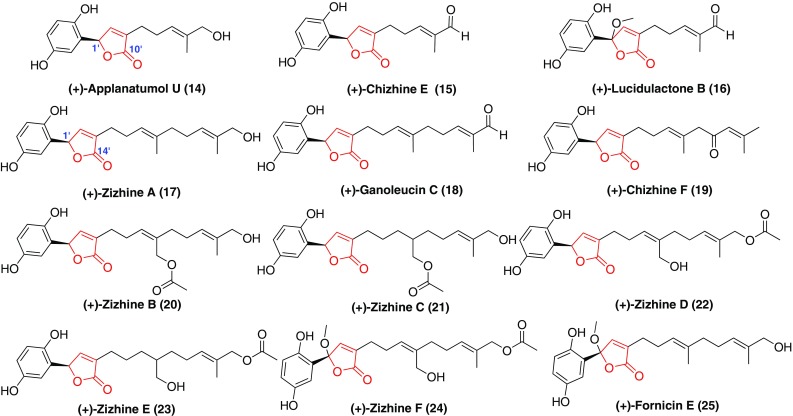
Structures of GMs with a γ-lactone

An *α*,*β*-unsaturated *γ*-lactone fraction can be formed through a nucleophilic reaction from the carboxyl at C-10′ or C-14′ to the ketone carbonyl at C-1′ (Fig. 2, Table 1). Cao et al [37] investigated the fruiting bodies of *G. sinense* and a series of GMs with an *α*,*β*-unsaturated *γ*-lactone fraction, namely (+)-zizhines A–F (**17**, **20**–**24**), were isolated. All the compounds were evaluated for their inhibition on extracellular matrix component (fibronectin) generation by using TGF-*β*1 induced rat kidney tubular epithelial cells. However, all of them didn’t show any inhibitory activities. (±)-Chizhine E and F (**15**, **19**) and (±)-lucidulactone (**16**) were isolated from *G. lucidum* and the individual enantiomers of compounds **15** and **19** significantly inhibit monocyte chemotactic protein 1 (MCP-1) and fibronectin production in a dose-dependent manner [31, 36]. Fornicin E (**25**) obtained from *G. capense* also was a pair of enantiomers, which showed stronger DPPH scavenging activity than vitamin E (positive control) [16]. (±)-Applanatumol U (**14**) was identified from *G. applanatum* and showed no inhibition against COX-1 and COX-2 [32].

**Fig. 3:**
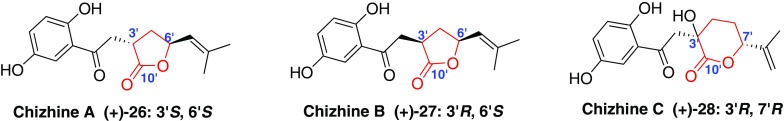
Structures of GMs with a (6′ → 10′)- or (7′ → 10′)-lactone

Three pairs of enatiomers (Fig. 3, Table 1), (±)-chizhines A–C (**26**–**28**) possessing a (6′ → 10′)-*γ*-lactone ring and a (7′ → 10′)-*δ*-lactone ring, respectively, were isolated from the fruiting bodies of *G. lucidum*. These compounds showed weak renoprotective effects [31].

**Fig. 4:**
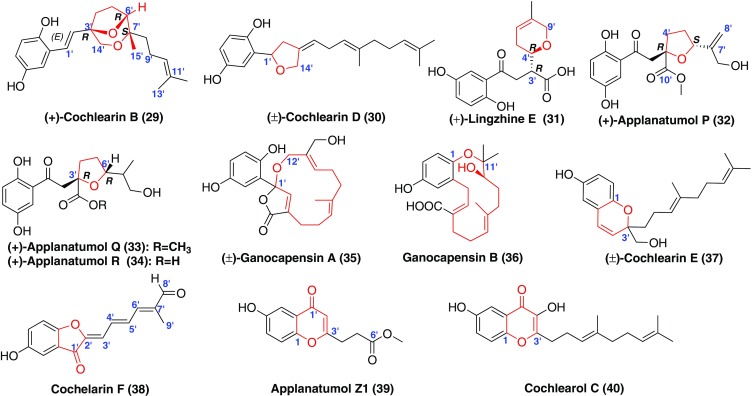
Structures of GMs with an ether ring

With the help of oxidases, the ether ring was present in many GMs (Fig. 4, Table 1). For example, compounds **29**–**35** had different ether ring in the terpenoid part, whereas, the ether rings in compounds **36**–**40** were formed through a cyclization between the hydroxyl at C-1 and the hydroxyls of the terpenoid part. Compounds **29**, **30**, and **35**–**38** displayed significant antioxidant activities in the DPPH scavenging assay [16, 30]. Among them, (±)-cochlearin D (**30**) and (+)-**30** exhibited weak inhibitory effects for the proliferation of hepatic stellate cells (HSCs) induced by transforming growth factor-*β*1 (TGF-*β*1) [30]. Except for above compounds, the rest of compounds didn’t show renoprotective activities [32, 33].

### Polycyclic GMs

Because of the presence of polyunsaturated terpenoid part, free radical reaction can be occurred in GMs under the conditions of enzyme and light, which led to the formation of polycyclic structures (Table 2).

**Table 2:** Name, source and bioactivities of polycyclic GMs

Number	Name	Bioactivity	Source	References
**41**	Applanatumol V	Inhibitory activities against COX-1 and COX-2	*G. applanatum*	[32]
**42**	Applanatumol W	Inhibitory activities against COX-1 and COX-2	*G. applanatum*	[32]
**43**	Applanatumol X	Inhibitory activities against COX-1 and COX-2	*G. applanatum*	[32]
**44**	Applanatumol Y	Inhibitory activities against COX-1 and COX-2	*G. applanatum*	[32]
**45**	Applanatumol Z	Inhibitory activities against COX-1, COX-2	*G. applanatum*	[32]
**46**	Applanatumol Z2	Inhibitory activities against COX-1, COX-2	*G. applanatum*	[32]
**47**	Applanatumol K	Inhibitory activities against COX-1 and COX-2	*G. applanatum*	[32]
**48**	Applanatumol L	Inhibitory activities against COX-1 and COX-2	*G. applanatum*	[32]
**49**	Applanatumol M	Inhibitory activities against COX-1 and COX-2	*G. applanatum*	[32]
**50**	Applanatumol N	Inhibitory activities against COX-1 and COX-2	*G. applanatum*	[32]
**51**	Applanatumol O	Inhibitory activities against COX-1 and COX-2	*G. applanatum*	[32]
**52**	Chizhiol A	Inhibitory activities against COX-1 and COX-2	*G. lucidum*	[39]
**53**	Ganotheaecoloid L	Inhibitory activities against COX-1 and COX-2	*G. theaecolum*	[40]
**54**	(+)-Ganotheaecoloid M	Inhibitory activities against COX-1 and COX-2	*G. theaecolum*	[40]
**55**	(−)-Ganotheaecoloid N	Inhibitory activities against COX-1 and COX-2	*G. theaecoloum*	[40]
**56**	Petchiene A	Inhibitory activities against COX-1 and COX-2	*G. petchii*	[41]
**57**	Lingzhine C	Promote proliferation of neural stem cells (NSCs)	*G. lucidum*	[38]
**58**	(±)-Lingzhine B	Inhibit NSC proliferation	*G. lucidum*	[38]
**59**	(−)-Ganotheaecoloid A	Inhibitory activities against COX-2	*G. theaecolum*	[40]
**60**	(−)-Ganotheaecoloid B	Inhibitory activities against COX-2	*G. theaecolum*	[40]
**61**	Ganotheaecoloid C	Inhibitory activities against COX-2	*G. theaecolum*	[40]
**62**	Ganotheaecoloid D	Inhibitory activities against COX-2	*G. theaecolum*	[40]
**63**	Ganotheaecoloid E	Inhibitory activities against COX-2	*G. theaecolum*	[40]
**64**	(−)-Ganotheaecoloid F	Inhibitory activities against COX-2	*G. theaecolum*	[40]
**65**	Ganotheaecoloid G	Inhibitory activities against COX-2	*G. theaecolum*	[40]
**66**	Ganotheaecoloid H	Inhibitory activities against COX-2	*G. theaecolum*	[40]
**67**	Ganotheaecoloid I	Inhibitory activities against COX-2	*G. theaecolum*	[40]
**68**	(+)-Ganotheaecoloid J	COX-2 inhibitory activity (IC_50_: 9.96 μM)	*G. theaecolum*	[40]
**69**	Ganotheaecoloid K	Inhibitory activities against COX-2	*G. theaecolum*	[40]
**70**	(+)-Cochlearin A	Antioxidant activity	*G. cochlear*	[30]
**71**	Spiroapplanatumine A	Inhibitory activities against JAK3 kinase	*G. applanatum*	[42]
**72**	Spiroapplanatumine C	Inhibitory activities against JAK3 kinase	*G. applanatum*	[42]
**73**	Spiroapplanatumine E	Inhibitory activities against JAK3 kinase	*G. applanatum*	[42]
**74**	Spiroapplanatumine G	Inhibitory activities against JAK3 kinase	*G. applanatum*	[42]
**75**	Spiroapplanatumine I	Inhibitory activities against JAK3 kinase	*G. applanatum*	[42]
**76**	Spiroapplanatumine B	Inhibitory activities against JAK3 kinase	*G. applanatum*	[42]
**77**	Spiroapplanatumine D	Inhibitory activities against JAK3 kinase (IC_50_: 7.0 ± 3.2 μM)	*G. applanatum*	[42]
**78**	Spiroapplanatumine F	Inhibitory activities against JAK3 kinase (IC_50_: 34.8 ± 21.1 μM)	*G. applanatum*	[42]
**79**	Spiroapplanatumine H	Inhibitory activities against JAK3 kinase	*G. applanatum*	[42]
**80**	Spiroapplanatumine J	Inhibitory activities against JAK3 kinase	*G. applanatum*	[42]
**81**	Spiroapplanatumine K	Inhibitory activities against JAK3 kinase	*G. applanatum*	[42]
**82**	Spiroapplanatumine L	Inhibitory activities against JAK3 kinase	*G. applanatum*	[42]
**83**	Spiroapplanatumine M	Inhibitory activities against JAK3 kinase	*G. applanatum*	[42]
**84**	(+)-Spiroapplanatumine N	Inhibitory activity against JAK3 kinase	*G. applanatum*	[42]
**85**	Spiroapplanatumine O	Inhibitory activities against JAK3 kinase	*G. applanatum*	[42]
**86**	(−)-Spiroapplanatumine N	Inhibitory activities against JAK3 kinase	*G. applanatum*	[42]
**87**	Spiroapplanatumine P	Inhibitory activities against JAK3 kinase	*G. applanatum*	[42]
**88**	Spiroapplanatumine Q	Inhibitory activities against JAK3 kinase	*G. applanatum*	[42]
**89**	(+)- Spirolingzhine A	Protective effects for NSC	*G. lucidum*	[38]
**90**	(+)-Spirolingzhine B	Protective effects for NSC	*G. lucidum*	[38]
**91**	(+)-Spirolingzhine C	Protective effects for NSC	*G. lucidum*	[38]
**92**	Spirolingzhine D	Protective effects for NSC	*G. lucidum*	[38]
**93**	(±)-Ganoderin A	Antioxidant activity	*G. cochlear*	[15]
**94**	Applanatumol H	Inhibitory activities against COX-1, COX-2	*G. applanatum*	[32]
**95**	Applanatumol I	Inhibitory activities against COX-1, COX-2	*G. applanatum*	[32]
**96**	Applanatumol J	Inhibitory activities against COX-1, COX-2	*G. applanatum*	[32]
**97**	Applanatumol D	Inhibitory activities against COX-1, COX-2	*G. applanatum*	[32]
**98**	Applanatumol E	Inhibitory activities against COX-1, COX-2	*G. applanatum*	[32]
**99**	Applanatumol J	Inhibitory activities against COX-1, COX-2	*G. applanatum*	[32]
**100**	Applanatumol F	Inhibitory activities against J COX-1, COX-2	*G. applanatum*	[32]
**101**	Lingzhilactone A	Renoprotective effect	*G. lucidum*	[43]
**102**	Lingzhilactone B	Renoprotective effect	*G. lucidum*	[43]
**103**	Lingzhilactone C	Renoprotective effect	*G. lucidum*	[43]
**104**	Applanatumol Z3	Inhibitory activities against JAK3 kinase	*G. applanatum*	[32]
**105**	Applanatumol Z4	Inhibitory activities against JAK3 and DDR1 kinases	*G. applanatum*	[32]
**106**	Applanatumol C	Inhibitory activities against JAK3 and DDR1 kinases	*G. applanatum*	[32]
**107**	(−)-Lingzhiol	Renoprotective effect	*G. lucidum*	[44]
**108**	(±)-Ganocochlearin A	Antioxidant activity	*G. cochlear*	[15]
**109**	(±)-Ganocochlearin B	Antioxidant activity	*G. cochlear*	[15]
**110**	(±)-Ganocochlearin C	Antioxidant activity	*G. cochlear*	[15]
**111**	(±)-Ganocochlearin D	Antioxidant activity	*G. cochlear*	[15]
**112**	Lingzhine D	Anti-BuChE activity	*G. lucidum*	[38]
**113**	(±)-Ganocin A	Anti-BuChE activity	*G. cochlear*	[45]
**114**	(±)-Ganocin B	Anti-BuChE activity	*G. cochlear*	[45]
**115**	(±)-Ganocin C	Anti-BuChE activity	*G. cochlear*	[45]
**116**	(±)-Ganocin D	Anti-BuChE activity	*G. cochlear*	[45]
**117**	Cochlearol A	Renoprotective effect	*G. cochlear*	[46]
**118**	Cochlearol B	Renoprotective effect	*G. cochlear*	[46]
**119**	Applanatumol A	Anti-renal fibrosis	*G. applanatum*	[18]
**120**	(±)-Applanatumol B	Anti-renal fibrosis	*G. applanatum*	[18]

**Fig. 5:**
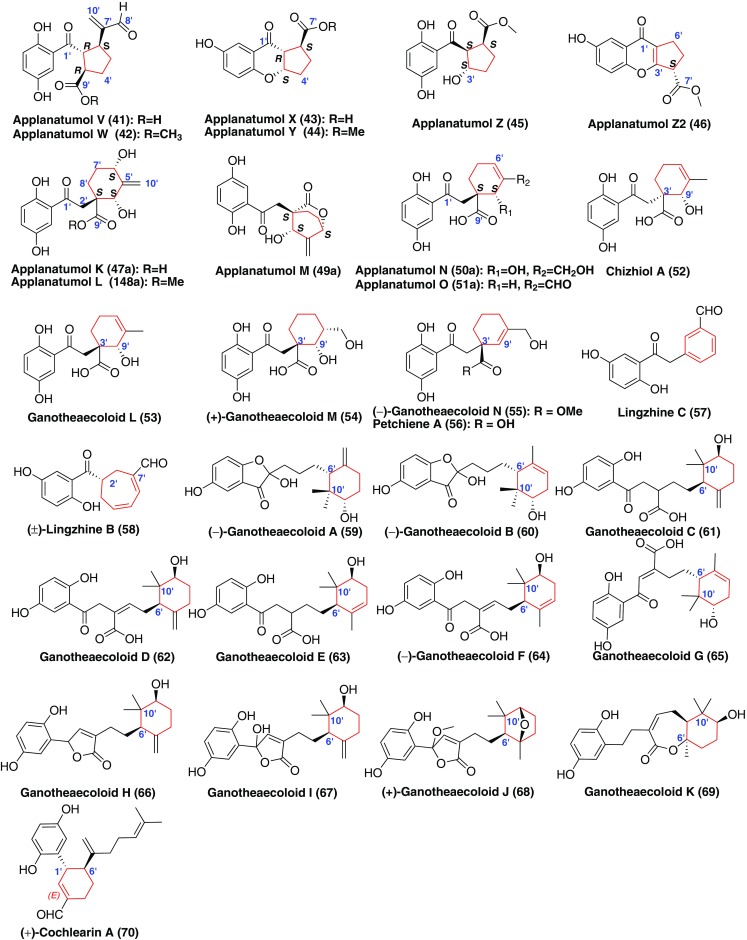
Structures of GMs with a five-membered or six-membered carbon ring

Compounds **41**–**58** (Fig. 5) were derived from the biogenetic precusor fornicin D (**1**), of which compounds **41**–**46** had a five-membered carbon ring in the terpenoid part through the connection between C-2′ and C-6′ [32]; wheares, compounds **47**–**57** possessed a six-membered carbon ring by a linkage between C-3′ and C-9′ [32, 38–41]. The presence of a seven-membered carbon ring in compound **58** was formed due to the carbon bond at C-2′ and C-9′ [38]. The inhibitory activities against COX-1 and COX-2 of compounds **41**–**56** were evaluated and they didn’t show obvious inhibition [32, 39–41]. Compound **57** was found to promote proliferation of neural stem cells (NSCs) [38]. However, compound **58** can inhibit NSC proliferation compared with a DMSO control [38].

When ganomycin C (**11**) was the biosynthetic precusor, compounds **59**–**70** (Fig. 5) were formed through the cyclization between C-6′ and C-10′ [30, 40]. Biological activity of all the GMs against COX-2 was evaluated in vitro, only ganotheaecoloid J (**68**) was found to have COX-2 inhibitory activity with an IC_50_ value of 9.96 μM [40]. Cochlearin A (**70**) showing DPPH scavenging activity had a cyclohexane fraction, which was formed by C-1′ binding with C-6′ [30].

**Fig. 6:**
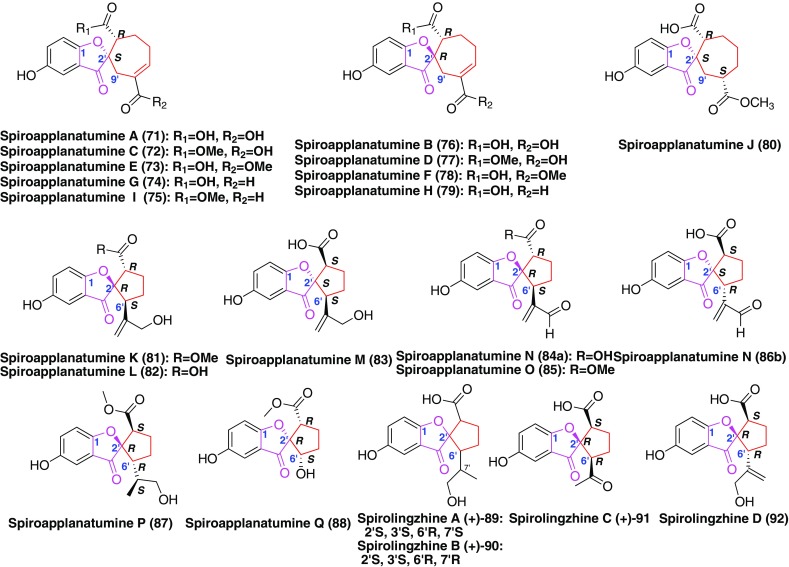
Structures of GMs with spiro ring

Furthermore, compounds bearing seven-membered carbon ring or five-membered carbon ring were as the precursor, the formation of an ether bond between C-1 and C-2′ resulted in the occurrence of sipro ring. For instance, compounds **71**–**80** (Fig. 6) contained a 6/5/7 ring system [42] and compounds **81**–**92** (Fig. 6) possessed a 6/5/5 ring system [38, 42]. Biological evaluation disclosed that compounds **77** and **78** inhibited JAK3 kinase with IC_50_ values of 7.0 ± 3.2 and 34.8 ± 21.1 μM, respectively [42]. The most potent member of this series, (−)-spirolingzhine A (**89**), was shown to affect NSC cell cycle progression using the 5-bromo-2-deoxyuridine (BrdU) incorporation assay [38].

**Fig. 7:**
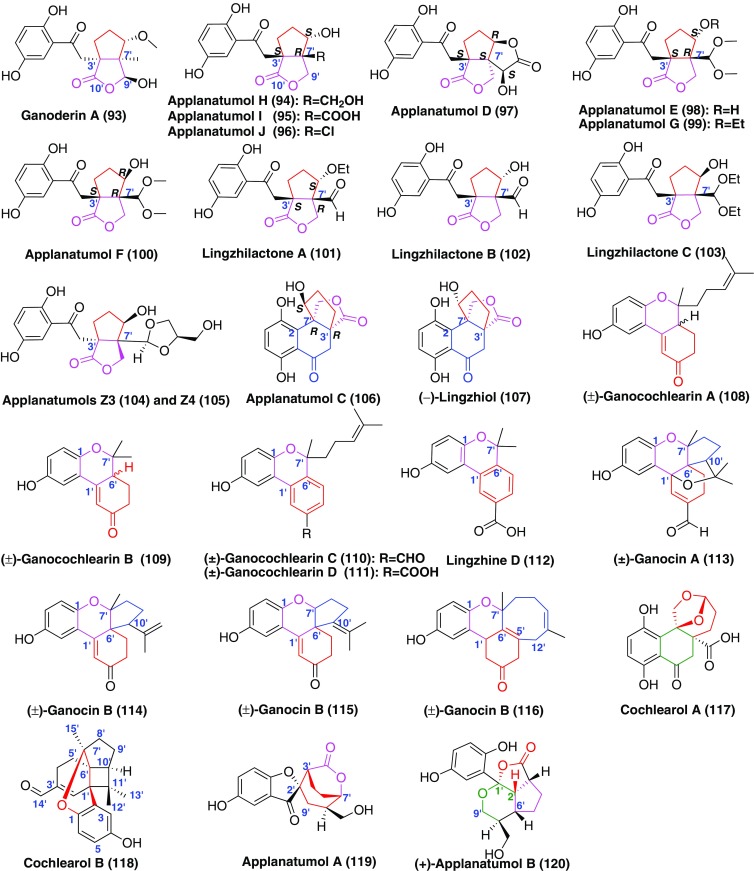
Structures of GMs with bridge ring

A series of bridge-ring compounds were formed through the free radical reactions. The structures of compounds **93**–**105** (Fig. 7) had a five-membered carbon ring fraction fusing with a *γ*-lactone ring [15, 32, 43]. Among them, ganoderin A (**93**) disclosed significant antioxdiant activities [15]. In the bioassay, compounds **94**–**100** didn’t exhibit inhibition aganist COX-1 and COX-2 [32]. The in vitro and in vivo results suggested that lingzhilactone B (**102**) could protect against renal injuries by increasing the activities of antioxidants and inhibiting inflammation [43]. The inhibition of Smad3 phosphorylation suggested that this substance displays in vivo antifibrotic activity by a mechanism that is dependent on disruption of Smad3. Applanatumol C (**106**) and linzhiol (**107**) beared an unusual 5/5/6/6 ring systerm characteristic of sharing a C-3′–C-7′ axis (Fig. 7) [32, 44]. The mirror of compound **106** was found to have COX-2 inhibitory effect with IC_50_ value of 25.5 mM [32]. (+)-Lingzhiol (**107**) and (−)-lingzhiol (**107**) could selectively inhibit the phosphorylation of Smad3 in TGF-*β*1-induced rat renal proximal tubular cells and activate Nrf2/Keap1 in mesangial cells under diabetic conditions [44].

Cochlearin A (**70**) was as the biogenetic precursor for compounds **108**–**112** (Fig. 7) with an additional ether bond (C-1–C-7′) [15, 38]. The further cyclization led to the formation of ganoderins A–C (**113**–**115**) (Fig. 7) possessing a spiro[4,5]decane ring system, along with ganocin D (**116**) (Fig. 7) with an eight-membered ring [45]. Similarly, compounds **108**–**112** showed comparable antioxidant effects compared to the positive control (Vitamin E) [15, 45], while compounds **113**–**116** (Fig. 7) displayed anti-BuChE activities [45]. Cochlearol A (**117**) was a new normeroterpenoid containing a naturally unusual dioxaspiro[4.5]decane motif [46]. Compound **118** (Fig. 7) was a novel meroterpenoid possessing respective 4/5/6/6/6 polycyclic ring systems [46]. Meanwhile, biological studies showed that (−)-**118** was a strong inhibitor of pSmads, exhibiting renoprotective activities in TGF-*β*1 induced rat renal proximal tubular cells [46]. Applanatumols A (**120**) and B [(±)-**121**] (Fig. 7) possessed a novel spiro[benzofuran-2,2′-biocyclo[3.2.2]nonane] ring system and a naturally unusual dioxacyclopenta[*cd*]inden motif, respectively [18]. Both of them didn’t show inhibitory activities against renal fibrosis in rat proximal tubular epithelial cells [18].

### Dimeric GMs

**Fig. 8:**
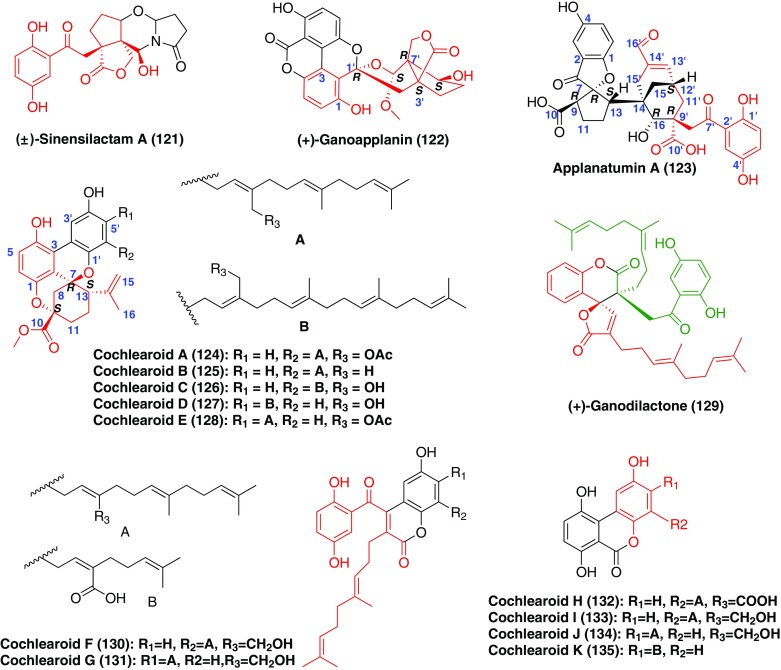
Structures of dimeric GMs

Except for the intramolecular cyclization, the intermolecular cyclization was present in GMs, which resulted in the formation of dimeric GMs (Fig. 8, Table 3). (+)- and (−)-siensilactam A (**121**) was a novel hybrid metabolites possessing a unique 2*H*-pyrrolo[2,1-*b*][1,3]oxaz-in-6(7*H*)-one ring system [47]. (−)-**121** was found to be a Smad3 phosphorylation inhibitor in TGF-*β*1 induced human renal proximal tubular cells [47]. (±)-Ganoapplanin (**122**) feartured an unprecedented dioxaspirocyclic skeleton, which was constructed from a 2,4-dihydroxy benzoic acid and a bridge-ring compound **102** [48]. Biological studies showed that (±)-**122** and its enantomers exhibited different inhibitory activities on T-tpye voltage-gated calcium channels [48]. Applanatumin A (**123**) possessed a new hexacyclic skeleton containing spro[benzofuran-2,1′-cyclopentane] motif [17]. The analysis of its sturcture showed that it consisted of two meroterpenoid parts, sproapplanatumine N (**84**) and applanatumol S (**5**), which were connected by a key Diels-Alder reaction. In TGF-*β*1-induced human renal proximal tubular cells, applanatumin A (**123**) diclosed potent antifibrotic activity [17]. Cochlearoids A–E (**124**–**128**) containing a unique methanobenzo[*c*]oxocino[2,3,4-*ij*]-isochromene scafflod were also constructed by two meroterpenoids [49]. Among them, (+)-**124**, and (−)-**126** significantly inhibited Cav3.1 TTCC and showed noticeable selectivity against Cav1.2, Cav2.1, Cav2.2 and Kv11.1 (hERG) channels [49]. The combination of two chian-contained GMs formed (+)-ganodilactone (**129**), cochlearoids F and G (**130** and **131**) [50, 51]. Similarly, when 2,4-dihydroy benzoic acid was linked with chain-contained GMs by the same method as ganoapplanin (**124**), compounds **132**–**135** were taken place. (±)-, (+)-, and (−)-ganodilactone (**129**) showed pancreatic lipase inhibitory activities and exhibited the IC_50_ values as 27.3, 4.0, and 2.5 μM, respectively [50]. In addition, other compounds were tested for their renoprotective activity against fibronectin inhibition in human proximal tubular epithelial cells (HKC-8). Compounds **130**–**133** and **135** exhibited potent inhibitory activity on fibronectin overproduction in TGF-*β*1-induced HKC-8 cells [51].

**Table 3:** Name, source and bioactivities of dimeric GMs

Number	Name	Bioactivity	Source	Reference
**121**	(−)-Sinensilactam A	Renoprotective activity	*G. sinense*	[47]
**122**	(+)-Ganoapplanin	Inhibitory activities on T-tpye voltage-gated calcium channels	*G. applanatum*	[48]
**123**	Applanatumin A	Antifibrotic activity	*G. applanatum*	[17]
**124**	(−)-Cochlearoid A	Inhibitory activities on T-tpye voltage-gated calcium channels	*G. cochlear*	[49]
**125**	(−)-Cochlearoid B	Inhibitory activities on T-tpye voltage-gated calcium channels	*G. cochlear*	[49]
**126**	(−)-Cochlearoid C	Inhibitory activities on T-tpye voltage-gated calcium channels	*G. cochlear*	[49]
**127**	(−)-Cochlearoid D	Inhibitory activities on T-tpye voltage-gated calcium channels	*G. cochlear*	[49]
**128**	(−)-Cochlearoid E	Inhibitory activities on T-tpye voltage-gated calcium channels	*G. cochlear*	[49]
**129**	(+)-Ganodilactone	Inhibitory activity against pancreatic lipase	*G. leucocontextum*	[50]
**130**	Cochlearoid F	Renoprotective effect	*G. cochlear*	[51]
**131**	Cochlearoid G	Renoprotective effect	*G. cochlear*	[51]
**132**	Cochlearoid H	Renoprotective effect	*G. cochlear*	[51]
**133**	Cochlearoid I	Renoprotective effect	*G. cochlear*	[51]
**134**	Cochlearoid J	Renoprotective effect	*G. cochlear*	[51]
**135**	Cochlearoid K	Renoprotective effect	*G. cochlear*	[51]

## Conclusion

In this review, we summarized the chemical structures and biological activities of 135 GMs in the last five years. Although the first GMs have been isolated in 2000, until recent years GMs were studied in-depth. Moreover, except for *G. lucidum* and *G. sinense* registered in Chinese Pharmacopoeia (2010 and 2015 edition), GMs were widely present in many other *Ganoderma* species, such as *G. appalantum*, *G. capense*, *G. cochlear*, and *G. petchii*. Above information indicated that GMs could play an important role in explaining the efficacy of *Ganoderma*. Thus, more bioactive studies should be carried out in the future for finding and developing lead compounds.

Furthermore, GMs possessed multiple prenyl groups or complex ring systems, which provided plentiful molecular model for various biological activities. However, we found that the majority of GMs showed racemic nature, which had impact on their bioactivites. Therefore, it is need to be separated using chiral HPLC method or be stereoselectively synthsized.

Addtionally, the formation of racemic GMs also attracted us attention. Analysis of these polycyclic GMs showed that their polycyclic structures are formed based on the polyunsaturated terpenoid fraction. Studies found that the cyclizations, such as cationic cyclization and radical cyclization, are the key factor to generate racemes. And these reactions can be taken place under conditions of acid, light and heating. However, the reactions in the plants mostly involved in enzyme system, which led to the generation of stereoselective compounds. Thus, we deduced that these polycyclic GMs with racemic nature may be formed for defending high temperature, strong light and diseases.

In all, the efforts to discover novel GMs with interesting biological activity and intriguing strutures from *Ganoderma* species have long been a hot topic in natural products chemistry. Meanwhile, novel GMs will serve as an abundant resource for synthetic chemists.
